# Tumoral Calcinosis Presentation in Operated Developmental Dysplasia of the Hip: A Case Report and Review of Literature

**DOI:** 10.7759/cureus.9948

**Published:** 2020-08-22

**Authors:** Mohammed H Al-Rumaih, Mohammed A Al-Otaibi, Abdulmuhsen N Alshammari

**Affiliations:** 1 Orthopaedic Surgery, Prince Sultan Military Medical City, Riyadh, SAU; 2 Orthopaedic Surgery, King Fahad General Hospital, Almadinah Almunawwarah, SAU

**Keywords:** calcification, complication, computed tomography (ct ), developmental dysplasia of the hip ( ddh ), tumoral calcinosis, magnetic resonance imaging (mri)

## Abstract

Tumoral calcinosis has long been a disputed clinical-pathological subject. It has been characterized by the deposition of calcium salt and hydroxyapatite in different periarticular soft tissue regions. It is most commonly seen in adults, and rarely seen in children. In this report, we present the case of a six-year-old girl referred to our institution for one year with a history of left hip pain and swelling. The patient underwent an open reduction of left hip and acetabuloplasty for developmental dysplasia of the hip (DDH) at the age of two years. Systematic investigations were performed and showed that the patient had abnormal calcifications and large, ill-defined lesions with an irregular margin on the left hip extended to the left gluteal area with skin ulceration suggestive of primary tumor calcinosis. Medical therapy has started, and a follow-up appointment was given to her in a pediatric metabolic clinic. In addition, we present a brief literature review of the effect of medical and surgical treatments on tumor calcinosis.

## Introduction

Tumoral calcinosis is considered a rare medical disorder characterized by the deposition of calcium salt and hydroxyapatite in different periarticular soft tissue regions [1]. It involved different regions such as hips, elbows, and shoulders, and they are the most commonly affected regions [2]. It can be divided into three types according to the mode of transmission and calcium-phosphorus metabolism imbalance. The first type is commonly seen in the adult population due to renal complications or calcium-phosphorus metabolism imbalance. The second type occurs due to the deposition of calcium, which results in skin ulceration lesion and infection. The mode of transmission could be familial or autosomal recessive. Patients can be presented either with hyperphosphatemia or normophosphatemia. The third type is characterized by normocalcemia and normophosphatemia. The patient can be presented before the second decade of life [3-5], and it has a sporadic pattern. The concept of tumoral calcinosis was described by Inclan et al. [6] in his review which defined this condition as an abnormal calcification of soft tissue. It occurs during the first or second decade of life. The site of lesion is close to large joints such as: hip, shoulder, and elbow. It is most commonly seen in the adult group and it is very rare in children. Diagnosis is based on the clinical finding, and imaging modalities such as: radiographic X-ray, CT, MRI, and histopathologic. It can mimic different medical disorders such as milk-alkali syndrome, hyperparathyroidism, hypervitaminosis D, chronic renal disease, osteochondroma, or chondrosarcoma [7]. It can be treated medically or surgically, and it depends on the clinical situation of the patient.

## Case presentation

**Figure 1:**
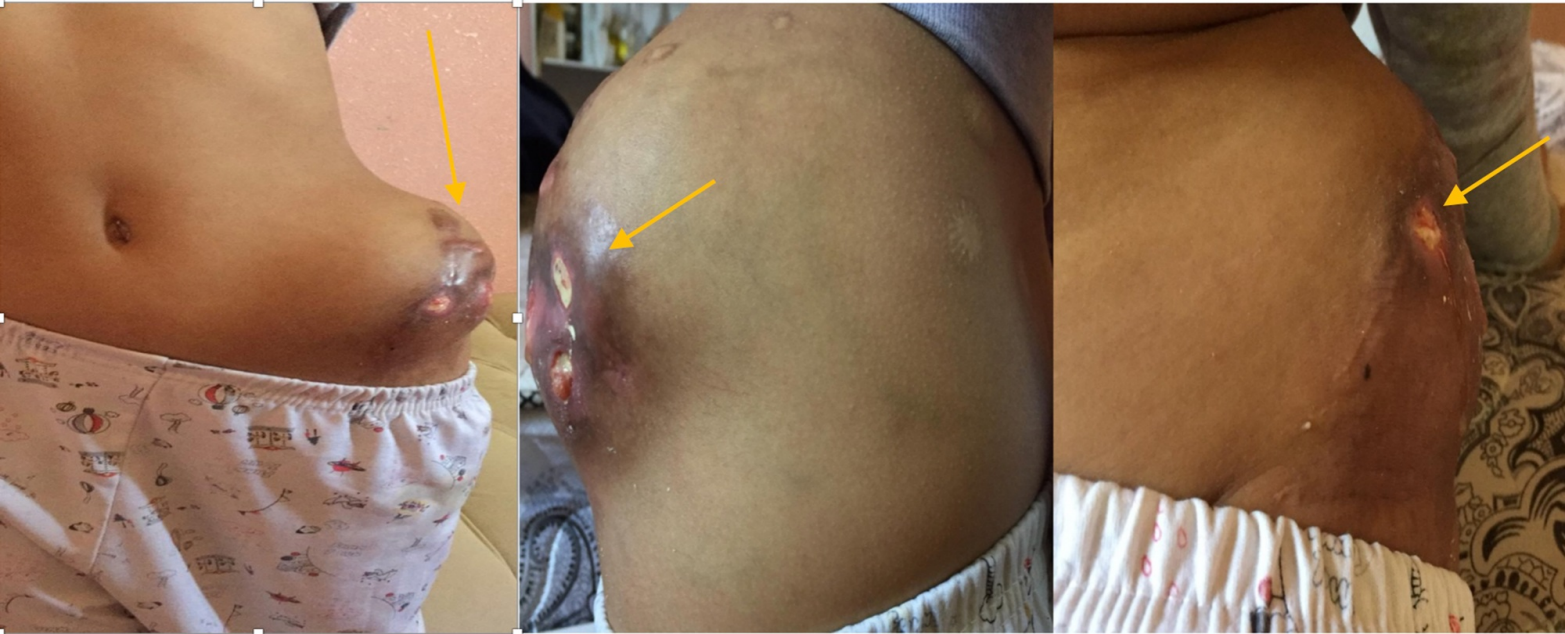
A large ill-defined lesion with irregular margin on the left hip extended to the left gluteal area with skin ulceration and exudates from the lesion.

**Figure 2:**
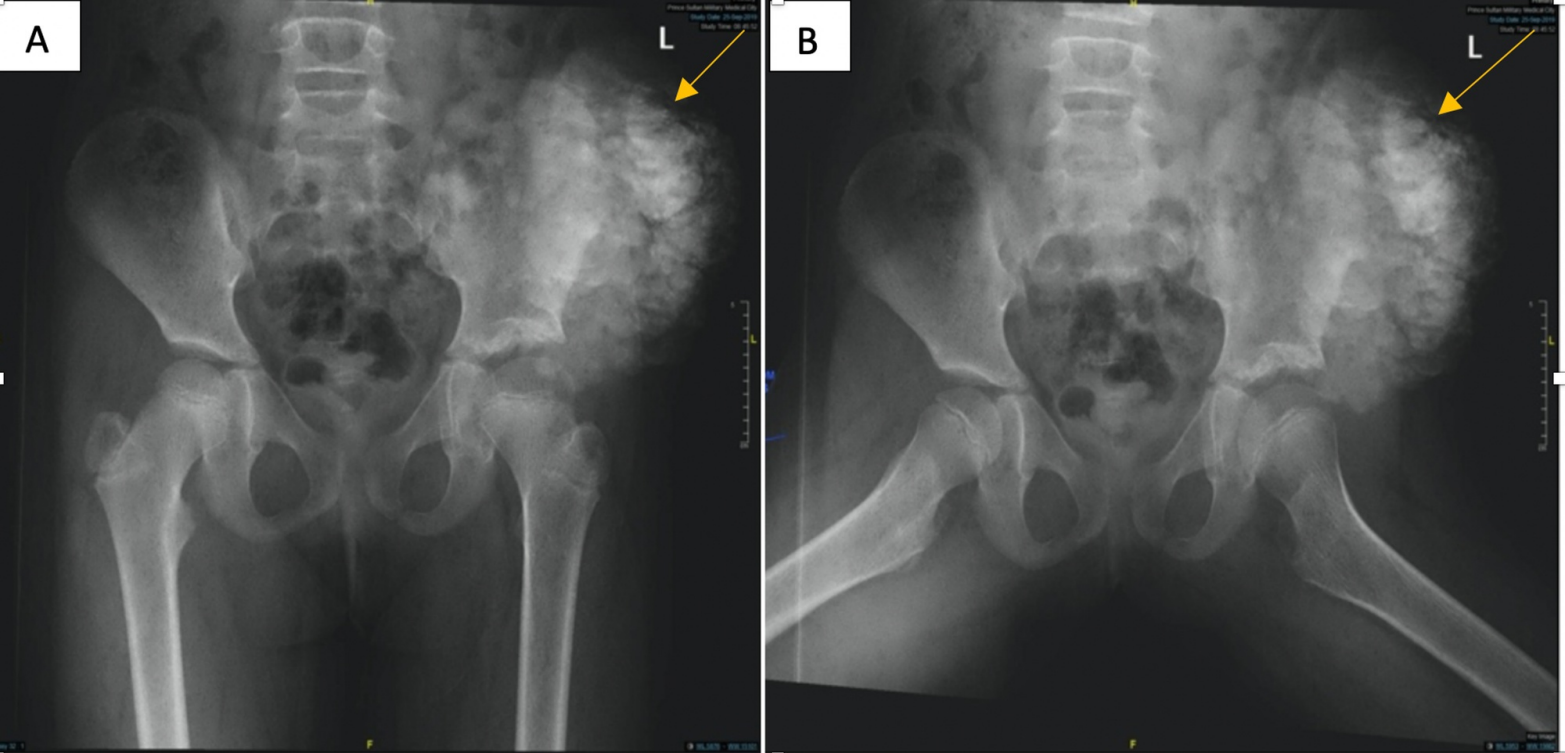
Radiographic pelvis X-ray, AP view "A" and Frog lateral view "B" showed large abnormal calcification on the left hip extended to the iliac bone. AP, anterior-posterior

A six-year-old girl was referred to our institution with a history of left hip pain and swelling for one year. The patient underwent for an open reduction of the left hip and acetabuloplasty for developmental dysplasia of the hip (DDH) at the age of two years. Upon examination, there was a mass on the left iliac bone extended to the gluteal area with skin ulcerations (Figure 1). Parents denied any similar presentation in their sibling as well as in the family. Radiographic pelvis X-ray was done for her, and it showed large abnormal calcifications on the left hip extended to the iliac bone (Figure 2A,B).

**Figure 3:**
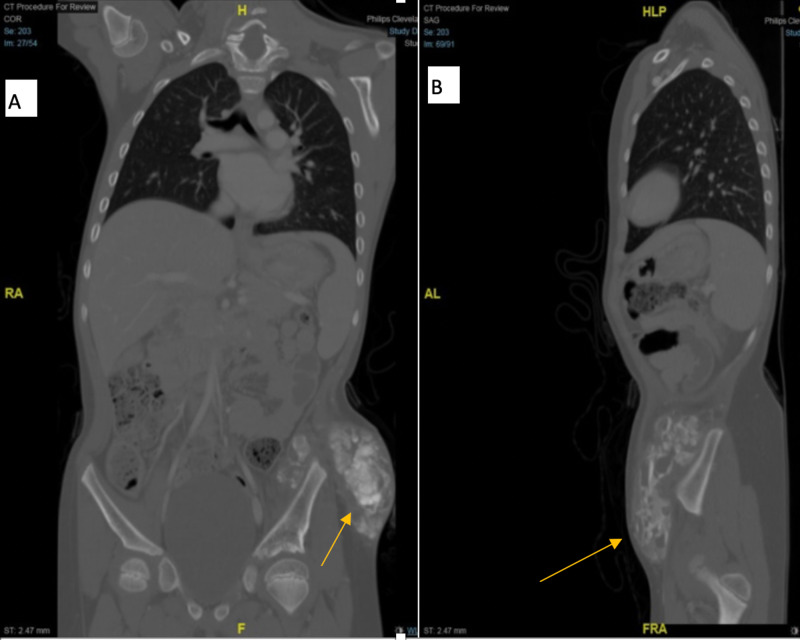
CT scan of the pelvis; coronal cut (A), and sagittal cut (B) showed large calcified lesion seen in the inner border of the left iliac bone extended laterally to the left lower abdominal wall with infiltration of the lateral abdominal wall. No evidence of bone destruction or periosteal reaction.

**Figure 4:**
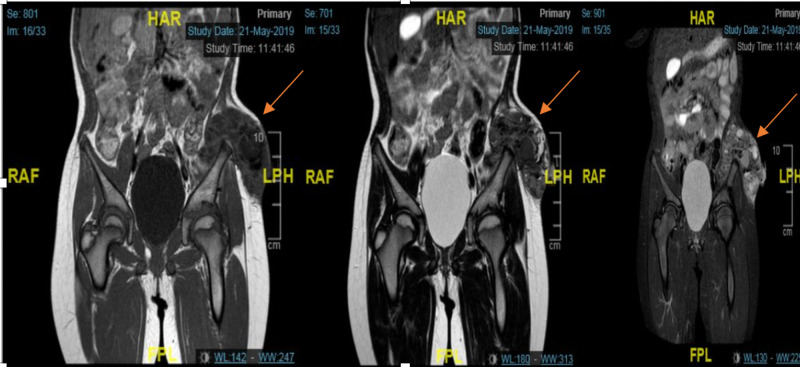
MRI of the pelvis showed a fairly defined cauliflower-like multilocular mainly single void sclerotic lesion with internal thick septations seen intimately related to inner surface of left iliac bone with no definite intraosseous origin. The lesion displays mixed low and intermediate single at T1 and mixed signals at T2.

Ultrasound of the pelvis showed a mixed echogenicity mass and pockets of a thick turbid collection with tiny calcifications. CT for the chest, abdomen, and pelvis were performed to exclude any malignancy. They showed a large calcified lesion (41 mm x 80 mm x 108 mm) seen in the inner border of the left iliac bone extended laterally to the left lower abdominal wall with infiltration of the lateral abdominal wall. The left iliac bone was intact with no evidence of bone destruction or periosteal reaction (Figure 3A,B). MRI was done as well, and it showed a fairly defined cauliflower-like multilocular, mainly a single void sclerotic lesion with thick internal septations (measures about 104 mm x 87 mm x 42 mm) seen intimately related to an inner surface of the left iliac bone with no definite intraosseous origin. The mass is seen fungating laterally through the lateral pelvic muscles to be subcutaneous and causes splaying of the lateral abdominal wall muscles and stretching of the overlying skin. The lesion displays mixed low and intermediate single at T1 and mixed signals at T2 (Figure 4).

The patient was referred to a pediatric metabolic clinic and they did for her a basic biochemical profile. They showed: white blood cell (WBC) level: 7.9 x 109/L (reference: 4.5-13.5 x 109/L), serum calcium: 2.56 mmol/L (reference: 2.17-2.51 mmol/L), serum phosphate: 1.15 mmol/L (reference: 0.95-1.75 mmol/L), alkaline phosphatase level: 159 U/L (reference: 142-335 U/L), erythrocyte sedimentation rate (ESR): 1 mL/h (reference: 0-15 mL/h), C-reactive protein (CRP): 23 mg/L (reference: 0-6 mg/L), Vitamin D: 19 pg/mL, and parathyroid hormone (PTH): 41 pg/mL (reference: 15-65 pg/mL). And the impression suggested primary tumoral calcinosis; they started her on medical therapy, and she was given a follow-up appointment in the pediatric metabolic clinic.

## Discussion

Medical and surgical treatment of tumoral calcinosis in children is still controversial, and they depend on the clinical situation of the patient. However, there are different literatures describing the efficacy of medical and surgical treatment in tumoral calcinosis in children. Sayar et al. [8] reported in his study of a four-year-old girl who presented to the pediatric surgery clinic due to swelling on the left gluteal region for three months. She had no history of trauma or infection. Radiographic pelvis X-ray showed abnormal calcifications on the left gluteal area that reached the femoral neck. Ultrasound showed hypoechogenicity on the left hip. Biochemical profiles were performed, and they showed the phosphate and alkaline phosphatase levels were elevated, whereas the calcium level was within normal limits. Pelvic MRI was done, and it revealed a nodular hypointensity lesion with subcutaneous fat tissue calcification at the left hip joint extended to the left gluteal area. Gluteal mass was excised without any complications, and the patient was given follow up after one year with no evidence of complications or recurrence. 

Kozlowski et al. [2] reported in his study about 13 cases of tumoral calcinosis in children. Three of those patients had abnormal calcifications at the hip joint. Both of them underwent surgical excision of the mass. One case had a recurrence after the surgical procedure, and the rest of the two patients were kept on a low phosphate diet, calcium diet, and aluminum hydroxide with no evidence of recurrence. Alam et al. [4] reported one case of a two-year-old boy who had a history of multiple nodular swellings over the right lower limb and right side of the chest. The patient had no history of trauma or infection. All of the biochemical profiles were within normal limits. Radiographic pelvis X-ray showed multiple ribbons like calcification of the soft tissue on the right hip. The biopsy was done, and it was revealed that multiple depositions of calcium extended to the dermis layer. The diagnosis was as a primary normophosphatemic tumoral calcinosis.

Bostrom [9] reported in his study a case of a five-month-old boy who was admitted under pediatric service due to bilateral knee swelling with large nodular lesion for the past three months. Biochemical profiles were within normal limits. Bone scan was done, and it showed high uptake in the soft tissue lesion anterior to both knees. At the age of seven months, they excised the mass without any complications. Two years after the surgery, the patient came to the clinic as a routine follow-up with no evidence of recurrence. 

Bittmann et al. [10] reported one case of a 15-year-old boy who presented with left gluteal swelling for three weeks. Histological examination confirmed the diagnosis of tumoral calcinosis, and the patient underwent surgical excision of the lesion with no evidence of complications or recurrence.

In addition, medical therapy has been shown to be an effective management in many literatures for tumoral calcinosis. Mitnick et al. [11] reported one case who had tumoral calcinosis of the hip, and they started him on calcium and phosphorus diets with diuretics for 22 days. The patient was evaluated and assessed based on serum and urine calcium, phosphorus levels, and it showed good improvement in terms of calcium and phosphorus homeostasis. Alkhooly [12] described in his study about four patients who had tumoral calcinosis in different sites of the body with skin ulceration. All of those patients had higher serum phosphorus and lower serum calcium levels. Phosphate-binding antacids and calcium-phosphorus diets were initiated for three years, and they followed them up to eight years after the treatment. It was showed the phosphorus level was dropped or near to normal range, and lesions were completely resolved in two patients with no complications or recurrence. All of those patients had full range of motion in their joints after medical therapy.

Mozaffarian et al. [13] reported in his study the effect of calcium-phosphorus diet with oral doses of aluminum hydroxide containing antacids in a patient with tumoral calcinosis, and they showed significant improvement in the clinical and radiological evaluation. Kirk and Simon [14] reported one case of tumoral calcinosis who had a large painful mass lesion on the shoulder with a limited range of motion. Phosphorus deprivation therapy showed marked improvement in treating of tumoral calcinosis.

## Conclusions

The clinical course of tumoral calcinosis and pathogenesis remains understood. The diagnosis of tumoral calcinosis is based on clinical evaluation, imaging modalities, and histopathologic examination. Treatment of tumoral calcinosis should be correlated with the patient and the clinical situation. Treatment options included both medical therapy and surgical intervention. It is better to start medical therapy in the early stage of the disease prior to surgical intervention. However, we need to conduct multiple large studies to show the effect of medical therapy in a patient with tumoral calcinosis.
